# Cell adhesion and growth enabled by biomimetic oligopeptide modification of a polydopamine-poly(ethylene oxide) protein repulsive surface

**DOI:** 10.1007/s10856-015-5583-3

**Published:** 2015-10-08

**Authors:** Jana Musilkova, Ilya Kotelnikov, Katarina Novotna, Ognen Pop-Georgievski, Frantisek Rypacek, Lucie Bacakova, Vladimir Proks

**Affiliations:** Department of Biomaterials and Tissue Engineering, Institute of Physiology of the Czech Academy of Sciences, v.v.i., Videnska 1083, 14220 Prague 4 - Krc, Czech Republic; Department of Biomaterials and Bioanalogous Systems, Institute of Macromolecular Chemistry, Academy of Sciences of the Czech Republic, v.v.i., Heyrovsky Sq. 1888/2, 16206 Prague 6, Czech Republic; Department of Chemistry and Physics of Surfaces and Biointerfaces, Institute of Macromolecular Chemistry, Academy of Sciences of the Czech Republic, v.v.i., Heyrovsky Sq. 1888/2, 16206 Prague 6, Czech Republic

## Abstract

Protein-repulsive surfaces modified with ligands for cell adhesion receptors have been widely developed for controlling the cell adhesion and growth in tissue engineering. However, the question of matrix production and deposition by cells on these surfaces has rarely been addressed. In this study, protein-repulsive polydopamine–poly(ethylene oxide) (PDA–PEO) surfaces were functionalized with an RGD-containing peptide (RGD), with a collagen-derived peptide binding fibronectin (Col), or by a combination of these peptides (RGD + Col, ratio 1:1) in concentrations of 90 fmol/cm^2^ and 700 fmol/cm^2^ for each peptide type. When seeded with vascular endothelial CPAE cells, the PDA–PEO surfaces proved to be completely non-adhesive for cells. On surfaces with lower peptide concentrations and from days 1 to 3 after seeding, cell adhesion and growth was restored practically only on the RGD-modified surface. However, from days 3 to 7, cell adhesion and growth was improved on surfaces modified with Col and with RGD + Col. At higher peptide concentrations, the cell adhesion and growth was markedly improved on all peptide-modified surfaces in both culture intervals. However, the collagen-derived peptide did not increase the expression of fibronectin in the cells. The deposition of fibronectin on the material surface was generally very low and similar on all peptide-modified surfaces. Nevertheless, the RGD + Col surfaces exhibited the highest cell adhesion stability under a dynamic load, which correlated with the highest expression of talin and vinculin in the cells on these surfaces. A combination of RGD + Col therefore seems to be the most promising for surface modification of biomaterials, e.g. vascular prostheses.

**Electronic supplementary material:**

The online version of this article (doi:10.1007/s10856-015-5583-3) contains supplementary material, which is available to authorized users.

## Introduction

Cell adhesion and proliferation are crucial steps in cell cultivation and in various tissue engineering applications. On most conventionally used biomaterials, such as synthetic polymers, ceramics or metals, the cell adhesion is mediated by proteins adsorbed on the material surface from biological fluids, including serum-supplemented cell culture media, blood or interstitial fluid. The amount, the types and the geometrical conformation of the adsorbed proteins is regulated by the physical and chemical properties of the material surface, e.g. its wettability, electrical charge and conductivity or the roughness and topography [1, 2]. However, the control of the cell behavior is not fully precise and not fully defined using this approach. In addition, the use of entire protein molecules can evoke an immune reaction after the modified material is implanted into the organism, if these molecules are of allogeneic or xenogeneic origin.

An alternative approach, which can circumvent these problems, is based on surface material coatings that completely suppress the non-specific protein adsorption and consequently cancel out the non-specific cell/material interactions. Surface adherent non-fouling layers can be achieved by a reaction of the polymer-functionalized terminal group with the material surface (“grafting to”), or by surface initiated polymerization from a surface-bound reactive species (“grafting from”). Some of the most effective non-fouling layers are realized utilizing dense poly(ethylene oxide) (PEO) [3] and ethylene oxide-based brushes, peptides [4], poly(betaine) zwitterions [5] and *N*-(2-hydroxypropyl)methacrylamide [6]. Protein-repulsive surfaces carrying reactive functional groups can easily be modified with various biomimetic groups (for example, the well-known fibronectin-derived RGD peptide ligand) [7]. Widely used bioconjugative reactions are exemplified by the thiolene reaction of thioles with an activated double bond [8], the alkyne-azide cycloaddition reaction [9] (click chemistry) and native chemical ligation [10].

On the one hand, this approach allows exact studies of primal cell adhesion, proliferation and differentiation. The adhesion ligands can be attached to biomaterials in defined types, concentrations, spacing, random or ordered distribution etc., which enable much more precise control of the number of adhering cells, their spreading area, proliferation, the switch between proliferation and differentiation, phenotypic maturation and cell functioning than the spontaneously adsorbed entire protein molecules. On the other hand, it has been shown that an increased concentration of adhesion ligands on the material surface decreased extracellular matrix (ECM) production by the cells adhering to these scaffolds, which limits the application of these scaffolds in tissue engineering [11]. The synthesis of ECM by these cells can be increased by tethering the transforming growth factor-beta (TGF-β) on the material surface [12] or by adding soluble TGF-β to the cell culture media [12]. However, protein-repulsive surfaces, used as a background for anchoring adhesion oligopeptides, allow no possibility or only a limited possibility of adsorbing the newly synthesized ECM molecules, even if their synthesis by cells is increased. This problem could be overcome by the use of oligopeptidic ligands not only to promote cell adhesion but also to anchor the newly synthesized ECM molecules to the material scaffolds.

In the present work, we therefore focused on the cultivation of vascular endothelial cells on non-fouling substrate-independent surfaces based on the polydopamine-poly(ethylene oxide) (PDA–PEO) system [13–16] allowing modification of any solid material. A combination of two different sorts of ligands was used for the biomimetic modifications. Specifically, fibronectin-derived ligands were used for integrin-mediated cell adhesion [17] and collagen-derived peptide ligands, responsible for the specific collagen–fibronectin interaction [18], were used to anchor cell-expressed fibronectin to the material surface. These ligands were applied in two concentrations, i.e. 90 or 700 fmol/cm^2^ in order to find their appropriate concentrations for cell adhesion, growth and matrix synthesis and deposition.

On the newly developed surfaces, functionalized either with RGD-containing oligopeptides, collagen-derived and fibronectin-binding peptides, or with combinations of both ligands, the adhesion, growth and expression of the gene for fibronectin were studied in vascular endothelial cells of the CPAE line. The cell behavior was investigated not only in the conventional static culture system but also in a dynamic culture system, better mimicking the conditions to which the cells are exposed in vivo, e.g. shear stress. Increased stability of the endothelial layer during the dynamic cultivation in our study was expected due to the additional binding to the cell-expressed fibronectin network anchored to the protein-repulsive surface.

## Materials and methods

Materials and methods used for the preparation of the model substrates, surface plasmon resonance (SPR) chips and cell seeding substrates are listed in Supporting Information.

### Peptide synthesis and radiolabeling

Azidopentanoyl-GGGRGDSGGGY-NH_2_ and azidopentanoyl-GGG-QRQVVGLOGQRGERGFOGLOG-NH_2_ (where O represents hydroxyproline) were prepared on solid phase using the standard Fmoc/tBu protocol, as described previously [19]. The azidopentanoyl-GGGRGDSGGGY-NH_2_ peptide for the model surface modification reaction was radiolabeled on solid phase according to Mackova et al. [20]. Peptide synthesis, peptide radiolabeling, substrate modification and a radioassay to determine the exact peptide surface concentration were performed according to Proks et al. [19]. The relationship between ligand distance and concentration was calculated according to hexagonal model proposed by Dalsin et al. [21].

### Preparation of the protein-repulsive surface

The protein repulsive substrates were prepared according to procedure described by Lee et al. [13]. The PDA coated surfaces were rinsed with copious amounts of H_2_O, were sonicated in H_2_O for 15 min to remove non-specifically bound microparticles and were dried in N_2_ gas. The PDA surfaces were stabilized by thermal annealing for 24 h at 110 °C before the PEO grafting procedure [22]. The average thickness of PDA–PEO layers determined by ellipsometry was 16.2 ± 3.1 nm for PDA and 12.7 ± 3.9 nm for PEO respectively. The same procedure was applied for the round microscopic glass coverslips (Menzel-Gläser, Germany, diameter 12 mm, #1, thickness 0.13–0.16 mm) for the cell culture studies. See Supporting Information for the detailed description of the procedure.

### Biomimetic modification of substrates

Biomimetic modification of the substrates was performed according to a previously described procedure [19]. Briefly, forty microliters of the reaction mixture containing peptide solution, copper sulfate and sodium ascorbate were dropped on Parafilm and were covered with the PEO-coated side of the glass slide. The substrates were incubated for 15 min and washed three times with *Milli-Q *water. The concentration of immobilized peptides was determined by a radioassay of ^125^I labeled azidopentanoyl-GGGRGDSGGGY-NH_2_ model reaction performed in the same condition.

The following groups of materials were prepared:

(1) A non-fouling PDA–PEO layer deposited on a glass coverslip (PEO),

(2) PEO modified with azidopentanoyl-GGGRGDSGGGY-NH_2_, referred to as “RGD”,

(3) PEO modified with 24 amino acid-long collagen-derived peptide sequence responsible for the collagen–fibronectin interaction (azidopentanoyl-GGG-QRQVVGLOGQRGERGFOGLOG-NH_2_), referred to as “Col”, and

(4) PEO modified with a combination of RGD + Col in a ratio of 1:1, i.e., one molecule of RGD per one molecule of Col.

Both peptides were used in two different concentrations, i.e. 90 and 700 fmol/cm^2^. Surfaces with a combination of RGD + Col contained 90 or 700 fmol/cm^2^ of each peptide. Unmodified microscopic glass coverslips (the same as used for the film deposition) and standard cell culture polystyrene wells (used for inserting the samples for cell cultivation) were used as reference materials. The glass coverslips were used preferentially for immunofluorescence studies due to their low autofluorescence and possibility to take microphotographs of cells at higher magnifications. As revealed by our preliminary studies, the cell behavior on both glass coverslips and polystyrene wells was similar.

### Cell seeding conditions

The materials were sterilized by UV light for 0.5 h from both sides. The samples were inserted into polystyrene 24-well cell culture plates (TPP, Trasadingen, Switzerland) and were seeded with endothelial cells originating from bovine pulmonary artery (line CPAE ATCC CCL-209, Rockville, MA, USA) in a minimum essential Eagle medium (E-MEM) supplemented with 2 mM l-glutamine, 1.0 mM sodium pyruvate, 0.1 mM non-essential aminoacids and 1.5 g/L sodium bicarbonate (all chemicals from Sigma). In order to avoid potential adsorption of serum-derived proteins and masking the oligopeptidic ligands, fetal bovine serum (Sebak GmbH, Aidenbach, Germany) was added to the medium 5 h after cell seeding to a final concentration of 20 % in each well [23]. Each well contained 30,000 cells and 1.5 ml of the medium. The cells were cultured in a humidified atmosphere containing 5 % CO_2_ in the air.

### Static cultivation conditions and evaluation of the cell number and morphology

On day 1, 3 and 7 after seeding, samples were placed in fresh 24-well plates, rinsed with phosphate-buffered saline (PBS; Sigma, USA), and the cells were detached from the materials by a trypsin-EDTA solution (Sigma, USA, Cat. No. T4174), and native unstained cells were counted in a Bürker haemocytometer. Three independent samples were used for each time interval and experimental group.

For evaluation of the shape and distribution of cells on the material surface, the cells were visualized by fluorescent dyes diluted in PBS, namely Hoechst #33342 (Sigma-Aldrich, 5 μg/ml), which stains the cell nuclei, and Texas Red C_2_-maleimide (Molecular Probes, Invitrogen, Cat. No. T6008, 20 ng/ml), which stains proteins of the cell membrane and cytoplasm. The cell pictures were taken using an epifluorescence microscope (IX 51, Olympus, Japan) equipped with a digital camera (DP 70, Olympus).

### Dynamic cultivation conditions and evaluation of the cell number

The cells were left to adhere for the first 24 h after seeding under static conditions, and then they were cultivated for 7 days under dynamic conditions using Mini Orbital Shaker SSM1 (Stuart), which provided a uniform circular motion. Thus, the endothelial cells were exposed on their top to a medium flow, which simulated, at least partly, the shear stress in blood vessels in vivo. The speed of the shaker was set to 50 rpm for the next 24 h, and was increased to 80 rpm for the rest of the cultivation period. Finally, the cells were counted in a Bürker haemocytometer. Three independent samples were used for each time interval and experimental group. Cell non-adhesive PEO surfaces were excluded from the evaluation.

The ratios of the cell numbers cultivated under static and dynamic conditions were also evaluated as a parameter describing the strength of cell adhesion, i.e. the ability of the adhering cells to withstand the dynamic load.

### Immunofluorescence staining

As markers of the formation of focal adhesion plaques on cells, talin and vinculin, i.e., important structural proteins present in focal adhesion plaques and associated with integrin adhesion receptors, were chosen. Talin binds to the intracellular β-subunit of integrin receptors and associates these receptors with vinculin. Vinculin then controls the focal adhesion formation by its direct interaction with actin cytoskeleton. This interaction leads to clustering of activated integrin receptors into focal adhesion plaques ([24]. Talin and vinculin were stained immunofluorescently on day 3 after seeding, using a procedure described earlier [23, 25]; see also the Supporting Information). Primary monoclonal antibodies (diluted 1:200 in PBS) were Anti-Talin, Clone 8d4 (Cat. No. T3287) and Anti-Vinculin, Clone hVIN-1 (Cat. No. V 9131), both from (Sigma, St. Louis, MO, USA). The secondary antibody was goat anti-mouse F(ab’)2 fragment of IgG conjugated with Alexa Fluor 488 (Molecular Probes, Eugene, OR; Cat. No. A11017; dilution 1:1000).

The fluorescence intensity on the microphotographs was measured using Fluorescence Image Analyser software (ver. 1.1, Matejka R., 2013, available from http://alice.fbmi.cvut.cz/software/fia). A single color plane threshold was set on each image to remove the non-protein area from the image data. These threshold and color plane settings were the same for each image of the protein that was measured. Then the cumulative sum of all pixel intensities was evaluated. The total immunofluorescence intensity of the protein was normalized per one cell. The number of analyzed cells was 5–19 cells for the 90 fmol/cm^2^ samples, and 17–24 cells for the 700 fmol/cm^2^ samples. Cell non-adhesive PEO surfaces were excluded from the evaluation.

The immunofluorescence pictures were also used for evaluating the spreading areas and the circumferences of the cells. The size of the area projected on the material was measured using Atlas software (Tescan Ltd., Brno, Czech Republic). Cells that developed intercellular contacts were excluded from the evaluation.

### Isolation of mRNA and qPCR

The cells were harvested after 3-day cultivation on the tested samples under static conditions. Total RNA was isolated using the Total RNA Purification Micro Kit (Norgen Biotek). The cells growing on the surfaces carrying oligopeptides were transferred in a fresh plate, rinsed with phosphate-buffered saline (PBS; Sigma, USA) and extracted from the material surface using the lysis solution enriched with 1 % mercaptoethanol. Five samples were pooled for the mRNA isolation. Reverse transcription was performed using Omniscript Reverse Transcription Kit (Qiagen).

The mRNA levels were quantified by quantitative real-time 5xHOT FIREPol Probe qPCR Mix Plus (ROX) (Solis BioDyne) and with TaqMan Gene Expression Assays (Life Technologies) labelled with FAM reporter dye specific to bovine TAL1 (Cat. No. Bt04291881_m1), VCL (Cat. No. Bt04306059_ m1) and FN1 (Cat. No. Bt00415008_m1). The experiments were performed in duplicates with ACTB (Cat. No. Bt03279174_m1, reporter dye VIC) as a reference gene, in a final reaction volume of 20 μl per well on a 96-well optical reaction plate using the Viia™ 7 Real-time PCR System.

Data are the mean of 4–5 experimental points from 2 independent experiments. Expression values were obtained from C_t_ numbers. The target gene levels are expressed as a relative value, the ratio of the target gene expression towards the reference ACTB gene. The relative gene expression was calculated as 2^−ΔCt^.

### Statistical analysis

The quantitative data was presented as mean ± standard error of mean (SEM). Statistical analyses were performed using SigmaStat (Jandel Corp., USA). Multiple comparison procedures were made by the one way analysis of variance (ANOVA), Student–Newman–Keuls method. *P* values equal to or less than 0.05 were considered significant.

## Results

### Fibronectin adsorption to the newly developed surfaces

**Fig. 1:**
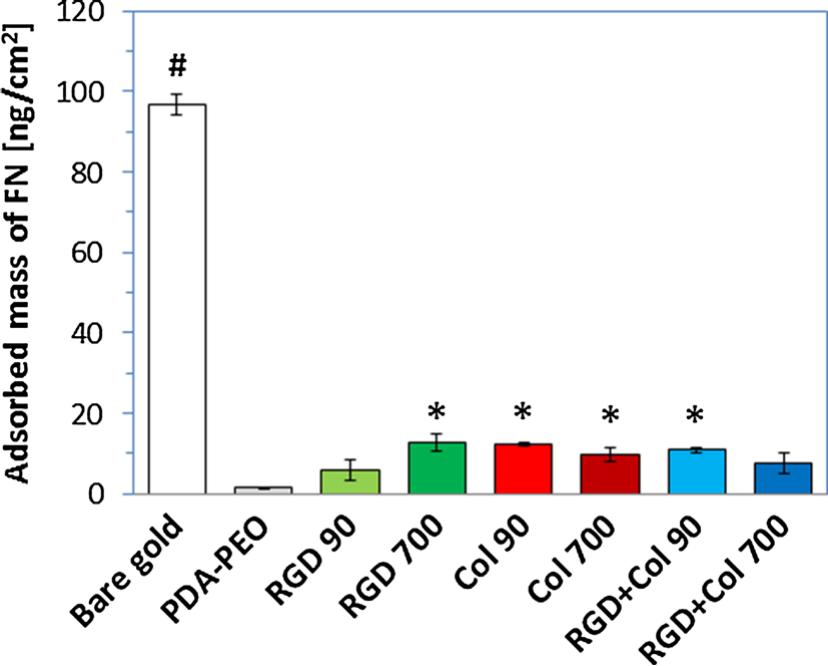
The adsorption of fibronectin (FN) on pristine PDA–PEO surfaces and these surfaces functionalized with RGD a collagen derived peptide (Col) or a combination of RGD + Col in concentrations of 90 or 700 fmol/cm^2^ for each peptide, as determined by SPR. FN adsorption on bare gold is reported as a reference. Mean ± SEM from 3 to 6 independent measurements. ANOVA Student–Newman–Keuls method. Statistical significance (*P* < 0.05): ^#^higher than all other groups, *higher than the PEO–PDA group

The short-term interaction between fibronectin (FN) and the pristine PDA–PEO and their counterparts bearing different peptide motifs were followed by SPR [26]. The pristine PDA–PEO layers proved to form an excellent barrier that almost completely cancels the nonspecific interactions between FN and the surface (Fig. 1). The FN deposits on these surfaces reached values of only 2 ng/cm^2^, which corresponds to a 98 % reduction compared to the deposits adsorbed on bare gold (97 ± 5 ng/cm^2^). Statistically significant (*P* < 0.05) deposits of FN on the majority of peptide bearing PEO–PDA layers were measured in comparison to the pristine PDA–PEO. Specifically, the adsorption of FN was significantly higher on surfaces with 700 fmol/cm^2^ of RGD, 90 and 700 fmol/cm^2^ of Col, and with 90 fmol/cm^2^ RGD + Col than on PDA–PEO, although this adsorption was significantly lower than on bare gold. Thus, the immobilization of the RGD, Col and RGD + Col peptide sequences decreased the initially observed non-fouling properties of the layers. The FN deposits on the peptide-bearing surfaces reached values of about 10 ng/cm^2^ (a reduction of 90 % in comparison with bare gold), irrespective of the peptide surface concentration, i.e. 90 or 700 fmol/cm^2^ (Fig. 1).

### Number of endothelial cells on the tested materials

**Fig. 2:**
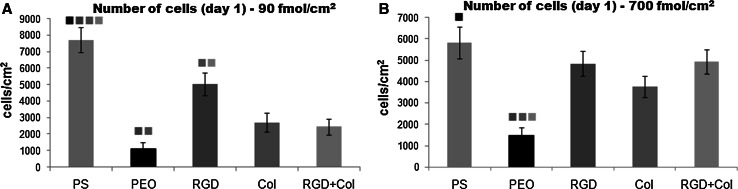
The number of endothelial CPAE cells initially adhering on standard cell culture polystyrene dishes (PS), on non-fouling PDA–PEO surfaces (PEO), and on PEO surfaces functionalized with RGD (RGD), a collagen derived peptide (Col) or a combination of RGD + Col in concentrations of 90 fmol/cm^2^ (**a**) or 700 fmol/cm^2^ (**b**) for each peptide. Mean ± SEM from three samples for each experimental group and time interval. ANOVA Student–Newman–Keuls method. Statistical significance (*P* < 0.05) in comparison with other experimental groups is indicated by the *colors* of these groups *above the columns* (Color figure online)

**Fig. 3:**
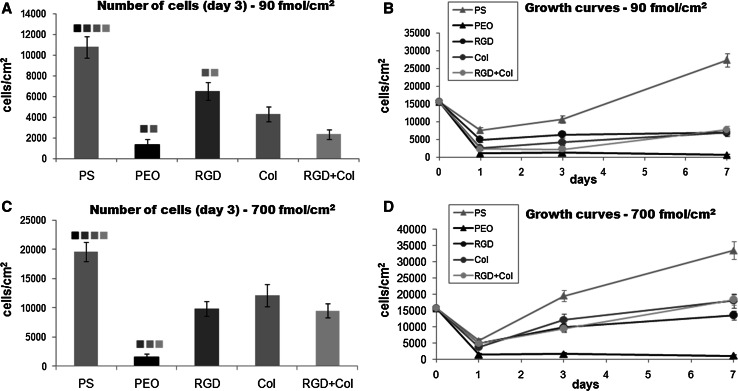
**a** and **c** The number of endothelial CPAE cells on day 3 after seeding on standard cell culture polystyrene dishes (PS), on non-fouling PDA–PEO surfaces (PEO), and on PEO surfaces functionalized with RGD (RGD), a collagen-derived peptide (Col), or a combination of RGD + Col in concentrations of 90 or 700 fmol/cm^2^ for each peptide. **b** and **d** Growth dynamics of endothelial cells during 1 week of cultivation on the same materials. Mean ± SEM from three samples for each experimental group and time interval. ANOVA, Student–Newman–Keuls method. Statistical significance (*P* < 0.05) in comparison with other experimental groups is indicated by the *colors* of the groups *above the columns*. **d** Col, RGD + Col: *P* < 0.05 compared to RGD on day 7 (Color figure online)

On day 1 after seeding, the numbers of initially adhered cells on pristine PEO-PDA surfaces, and also on all surfaces modified with oligopeptides at the concentration of 90 fmol/cm^2^, were significantly lower than on the standard cell culture polystyrene dishes (Fig. 2a). Only the cell numbers on surfaces with RGD or Col were significantly higher than the cell numbers on PEO-PDA. However, on surfaces functionalized with oligopeptides in the concentration of 700 fmol/cm^2^, the cells reached significantly higher numbers than on PEO-PDA, and these numbers approached the value on the control polystyrene (Fig. 2b). Similar cell behavior on the tested surfaces was also observed on day 3 after seeding (Fig. 3a, c). On day 7 after seeding, the cell numbers were higher on the peptide-bearing surfaces than on the pristine PEO-PDA surfaces, but these numbers still remained lower than on the control polystyrene dishes. However, from day 3 to 7, the cells on the RGD + Col surfaces with both peptide concentrations proliferated more quickly than the cells on the RGD surfaces, as indicated by the steeper rise of the growth curves (Fig. 3b, d). As a result, the cells on surfaces with RGD + Col concentration attained a similar or a slightly higher number in comparison with the other peptide-modified surfaces.

### Ratio of the cell numbers under dynamic and static cultivation

**Fig. 4:**
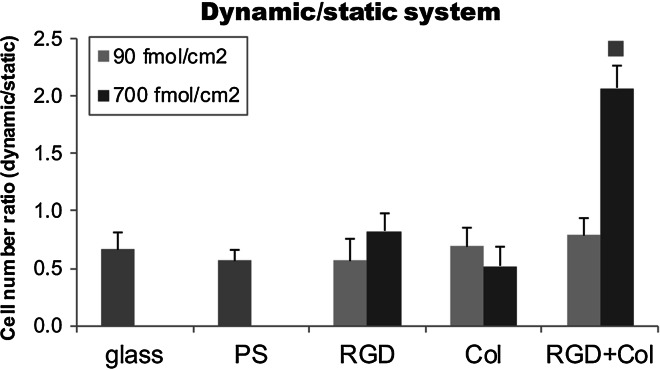
The ratio of cell numbers obtained in dynamic and static cell culture systems on day 7 after seeding on microscopic glass coverslips (glass), on standard cell culture polystyrene dishes (PS), and on PEO surfaces functionalized with RGD (RGD), with a collagen-derived peptide (Col) or with a combination of RGD + Col in concentrations of 90 fmol/cm^2^ (*light blue columns*) or 700 fmol/cm^2^ (*dark blue columns*) for each peptide. Mean ± SEM from 53 samples for each experimental group. ANOVA, Student–Newman–Keuls method. Statistical significance: ^■^ *P* < 0.05 compared with all other samples (Color figure online)

Interesting results were obtained when the cells were cultivated under dynamic conditions using the Mini Orbital Shaker SSM1. In this system the medium flows over the endothelial cells, exerting a shear stress on them. On day 7 after seeding, the ratio between the cell numbers in the dynamic and conventional static culture systems was calculated. This number can be considered as a measure of the resistance of the cells to detachment by the dynamic load. At a lower peptide concentration, this resistance was similar on all tested samples, i.e. on microscopic glass coverslips, on polystyrene dishes, and on samples functionalized with RGD, Col or RGD + Col. However, at a higher peptide concentration, the resistance was remarkably higher on the RGD + Col surfaces than on the other samples (Fig. 4). Thus, RGD-functionalization seems to be important for initial cell adhesion, but combined RGD + Col functionalization is necessary for further growth and retention of the cells, particularly under a dynamic load.

### Morphology and spreading of endothelial cells cultured on the tested materials

**Fig. 5:**
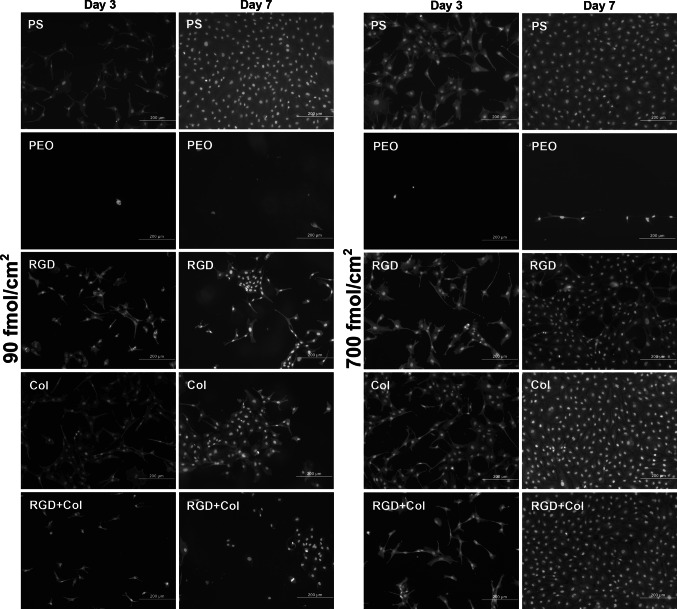
Morphology of endothelial CPAE cells on days 3 and 7 after seeding on standard cell culture polystyrene dishes (PS), on non-fouling PDA–PEO surfaces (PEO), and on PEO surfaces functionalized with RGD (RGD), with collagen-derived peptide (Col) or with a combination of RGD + Col at concentrations of 90 or 700 fmol/cm^2^ for each peptide. Cells stained with Texas Red C_2_-Maleimide and Hoechst #33342. Olympus IX 51 microscope, obj. ×20, DP 70 digital camera, *bar* 200 µm

**Fig. 6:**
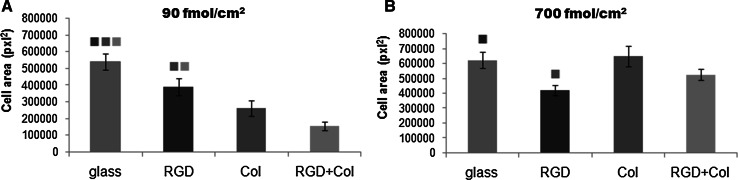
The size of the spreading areas of the endothelial CPAE cells on day 3 after seeding on the control microscopic glass coverslips (glass), PEO surfaces functionalized with RGD, with collagen-derived peptide (Col) or with a combination of RGD + Col in a concentration of 90 fmol/cm^2^ (**a**) or 700 fmol/cm^2^ (**b**) for each peptide. Measured on pictures of cells with immunofluorescence staining of talin and vinculin. Mean ± SEM from 5 to 19 measured cells for the 90 fmol/cm^2^ samples, and 17–24 cells for the 700 fmol/cm^2^ samples. ANOVA, Student–Newman–Keuls method. Statistical significance (*P* < 0.05) in comparison with other experimental groups is indicated by the *colors* of the groups *above the columns* (Color figure online)

On the PEO–PDA layer, the cells were very sparse and round, and if they were spread, it seemed that they were located in defects of the PEO films, such as scratches caused by handling the samples (Fig. 5). On the other hand, the cells on the peptide-functionalized surfaces were more numerous, well-spread and mostly polygonal, although the number and spreading of cells on samples with the lower concentration of Col and RGD + Col were lower than on polystyrene and glass coverslips (Figs. 5, 6a). On samples with a higher concentration of Col and RGD + Col, the cell spreading area on day 3 after seeding was similar to that on control glass coverslips (Fig. 6b), and on day 7 (Fig. 6), the cells on these samples formed fully confluent layers similarly as on polystyrene (Fig. 5). Only on surfaces with RGD, the cell spreading area was significantly smaller than on glass (Fig. 6b), and on day 7, these cells were subconfluent (Fig. 5).

### Immunofluorescence of talin and vinculin in cells on the tested materials

**Fig. 7:**
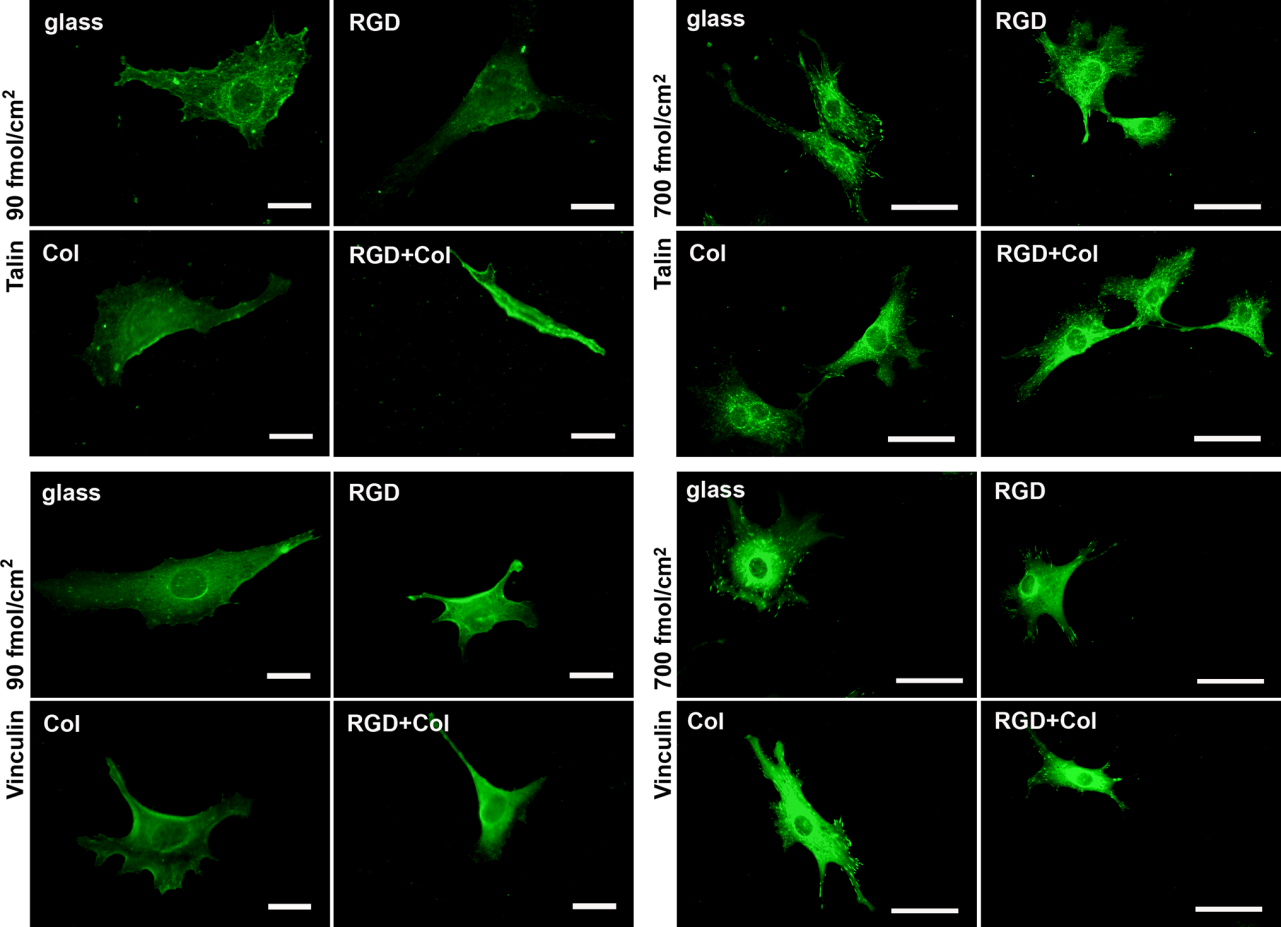
Immunofluorescence staining of talin and vinculin in endothelial CPAE cells on day 3 after seeding on the control microscopic glass coverslips (glass), and on PEO surfaces functionalized with RGD, with a collagen-derived peptide (Col) or with a combination of RGD + Col in concentrations of 90 or 700 fmol/cm^2^. Magnification, *bar*, exposure time: 90 fmol/cm^2^ samples: ×64, 20 µm, talin: 1800 ms, vinculin: 1400 ms; 700 fmol/cm^2^ samples: ×40, 50 µm, talin: 909 ms, vinculin: 625 ms, respectively

**Fig. 8:**
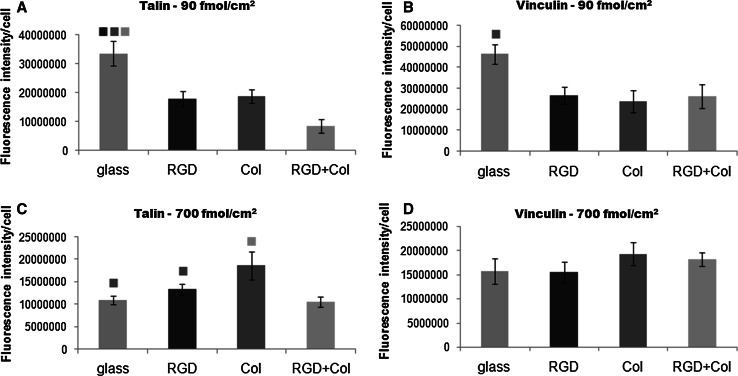
Intensity of the immunofluorescence of talin (**a**, **c**) and vinculin (**b**, **d**) in endothelial CPAE cells on day 3 after seeding on the control microscopic glass coverslips (glass), on PEO surfaces functionalized with RGD (RGD), with a collagen-derived peptide (Col) or with a combination of RGD + Col in concentrations of 90 fmol/cm^2^ (**a**, **b**) or 700 fmol/cm^2^ (**c**, **d**) for each peptide. The magnification and exposure time of pictures used for the measurement is described in Fig. 7. Mean ± SEM from 5 to 19 cells for 90 fmol/cm^2^ samples, and 17–24 cells for 700 fmol/cm^2^ samples. ANOVA, Student–Newman–Keuls method. Statistical significance (*P* < 0.05) in comparison with other experimental groups is indicated by the *colors* of the groups *above the columns* (Color figure online)

Talin- or vinculin-containing focal adhesion plaque were more numerous and better-developed in cells on surfaces with a higher peptide concentration and on the control microscopic glass coverslips than in the cells on surfaces with a lower peptide concentration, where talin and vinculin were distributed rather diffusely (Fig. 7). Similarly, the intensity of the fluorescence of talin and vinculin in the cells on surfaces with a lower peptide concentration was generally lower than in the cells on the glass coverslips (Fig. 8a, b). On the samples with a higher peptide concentration, the intensity of the fluorescence of talin and vinculin was usually similar to the value detected in the cells on the glass coverslips (Fig. 8c, d). Moreover, in the cells on samples with a Col concentration of 700 fmol/cm^2^, the intensity of the fluorescence of talin was even higher than on the glass coverslips and on surfaces with the corresponding concentrations of RGD and RGD + Col (Fig. 8c).

### Expression of genes for talin, vinculin and fibronectin in cells on the tested surfaces

**Fig. 9:**
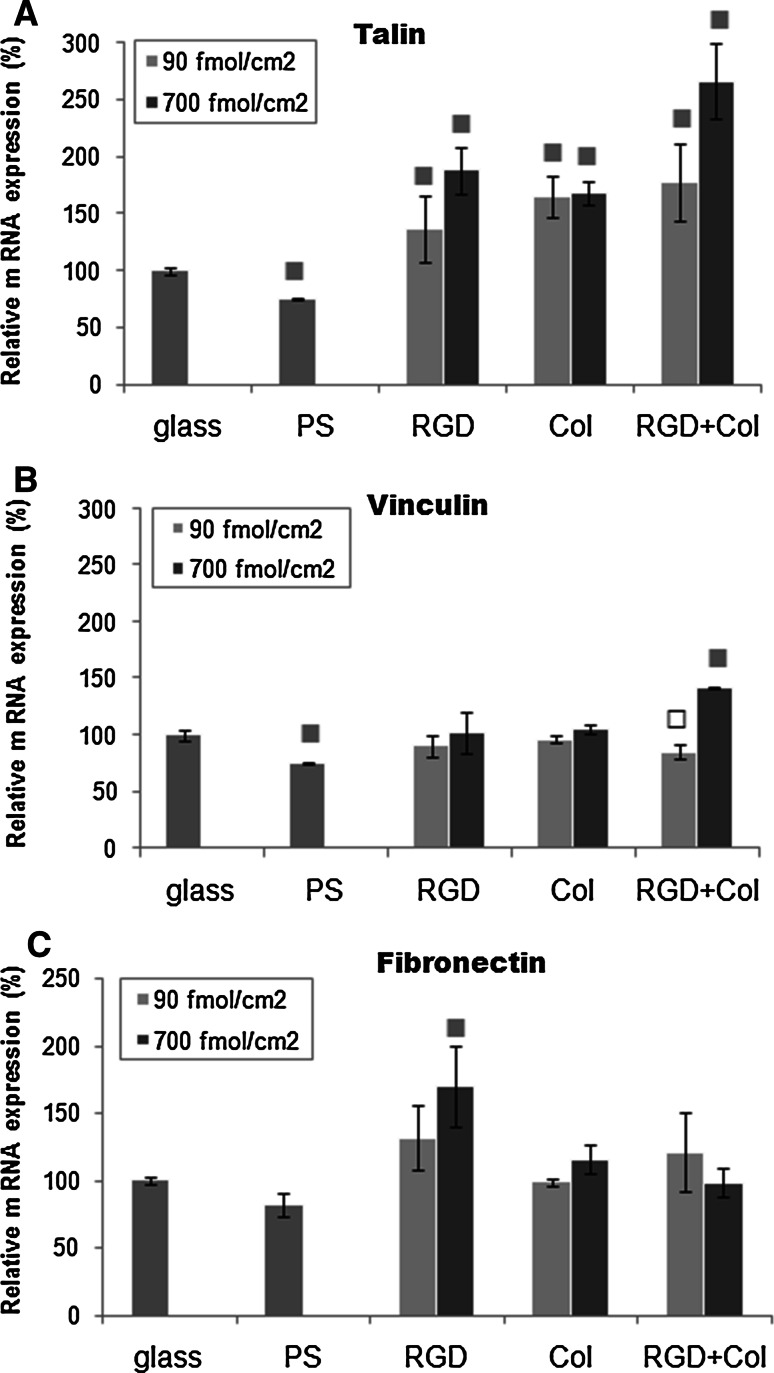
Relative expression of genes for focal adhesion proteins talin (**a**), vinculin (**b**) and for an ECM protein fibronectin (**c**) in endothelial CPAE cells on day 3 after seeding on the control microscopic glass coverslips (glass), on standard polystyrene cell culture dishes (PS), and on PDA–PEO surfaces functionalized with RGD (RGD), with a collagen-derived peptide (Col) or with a combination of RGD + Col peptides in concentrations of 90 or 700 fmol/cm^2^ for each peptide. The data are expressed as values relative to the gene expression in cells grown on microscopic glass coverslips (100 %). Mean ± SD. (Standard deviation) from 5 to 6 experimental points from three independent experiments. ANOVA, Student–Newman–Keuls method. Statistical significance: ^■^ *P* < 0.001, ^□^ *P* < 0.02 compared to the values on glass

We found that the expression of talin in cells growing on the surfaces bearing all peptides in both concentrations was significantly higher than in the cells on the control glass coverslips and on polystyrene dishes. The maximum talin expression was achieved in cells growing on surfaces with RGD + Col in a concentration of 700 fmol/cm^2^ (Fig. 9a). The relative mRNA expression of talin in the cells on this sample reached 268 % of the value obtained on the control glass coverslips. This result was in good correlation with the highest resistance of cells on these samples to the dynamic load (cf. Fig. 4). In addition, the expression of vinculin was significantly higher in the cells on surfaces with RGD + Col in a higher concentration than on the glass coverslips and polystyrene dishes, while in the cells on samples with RGD or Col only, the vinculin expression was similar as in the cells on both control substrates (Fig. 9b). Surprisingly, there was the highest expression of fibronectin in the cells grown on surfaces functionalized with RGD (700 fmol/cm^2^), though this had been anticipated on surfaces containing the fibronectin-binding collagen-derived peptide (Col, RGD + Col).

## Discussion

Protein-repulsive surfaces have been widely used for modulating cell adhesion and proliferation. The non-fouling properties of these surfaces have been exploited for constructing biochips [27], for antimicrobial coatings [28], for inhibiting platelet adhesion and hemocoagulation on blood-contacting devices [29], for functionalization with growth and differentiation factors [12, 30, 31] and particularly as a cell non-adhesive bioinert background for the attachment of adhesion oligopeptides in defined concentrations, spacing and distribution in order to control the adhesion, spreading, growth and differentiation of cells [19, 23, 25, 32]. Our recent work investigated the level of protein absorption on an RGD-modified PDA–PEO surface (unpublished results). The amino acid sequence RGD is typically contained in fibronectin and vitronectin, and is recognized by integrin adhesion receptors on many cell types. The novelty of this study lies in the modification of the material surface not only with RGD as a ligand for cell adhesion receptors, but also with ligands allowing a specific interaction with fibronectin in order to facilitate the deposition of ECM on the scaffolds. We hypothesized that immobilizing the collagen fragment responsible for collagen–fibronectin interaction allows cell-expressed fibronectin to be specifically anchored to the protein-repulsive surface. The collagen I-derived peptide QRQVVGLOGQRGERGFOGLOG-NH_2_ was chosen for these purposes [18].

The peptide concentration level on the material surface was adjusted according to the following assumption. On the one hand, the maximal peptide surface concentration should be at a level where the only peptide ligand can interact with the only integrin receptor. Adopting the published diameter of the integrin head of 7–10 nm [33], we deduced that the ligand surface concentration must be below 2000 fmol/cm^2^.

On the other hand, the minimal peptide concentration for integrin-mediated cell adhesion has been observed at a level of 10 fmol/cm^2^ [34], and the optimal RGD spacing distance for fibroblast focal adhesion formation has been observed at a level of 58 nm, which represents a peptide surface concentration of 57 fmol/cm^2^ [35].

We therefore decided to use a peptide ligand concentration at levels of 1000 and 100 fmol/cm^2^. For 12.7 nm in thickness, the concentration of the alkyne group suitable for peptide modification is at a level of 2.76 × 10^−10^ mol/cm^2^. To achieve the designed peptide surface concentration, the sample modification was therefore driven using diluted peptide solutions according to our earlier study [19].

The real level of 90 and 700 fmol/cm^2^ of the ligand surface concentration was finally determined by a radioassay of the ^125^I labeled peptide, which corresponds to ligand spacing of 46 and 14 nm, respectively. For surfaces modified by a combination of RGD + Col, containing 90 and 700 fmol/cm^2^ for each peptide, i.e. 180 and 1400 fmol/cm^2^ in total, the ligand spacing was shorter, i.e. 33 and 10 nm, respectively.

The first finding of this study confirmed the cell non-adhesive effect of the protein-repulsive PEO layer. As is generally known, this effect is due to the high hydrophilicity of this layer, associated with the mobility of the densely packed PEO chains, which hinders protein adsorption and successive cell adhesion. Similar results were also obtained in our earlier study performed on rat aortic smooth muscle cells cultured on PDLLA–PEO copolymers with 33 % of PEO [25]. Even on peptide-functionalized surfaces, the adsorption of fibronectin was very low, and was similar in all peptide-bearing surfaces. Surfaces exhibiting such low fouling from fibronectin can be expected to elicit different peptide sequences, so it was possible to establish the specific cell/RGD-ligand and cell/FN/Col in the cell seeding studies.

When the PEO surfaces were functionalized with higher concentrations of RGD, with a collagen-derived peptide (Col), or with a combination of RGD + Col, cell adhesion and growth were almost completely restored, i.e. became similar as on standard cell culture polystyrene dishes or on control microscopic glass coverslips. However, on surfaces with a low peptide concentration, the cell colonization was noticeably improved practically only on the RGD-functionalized surface. The cell numbers on the RGD + Col surface even did not significantly exceed the values on the PEO surface, and the spreading of cells on RGD + Col and Col surfaces was significantly lower than on RGD-bearing surfaces. These results were surprising, because the ligand spacing on the Col and RGD + Col samples with a lower ligand concentration was calculated to be 46 and 33 nm, which is below the limit of 70 nm ligand spacing described for the spreading and growth of mouse osteoprogenitor MC3T3-E1 cells and rat bone marrow mesenchymal stem cells [36, 37].

An explanation for this cell behavior could therefore lie in differences in binding the RGD and Col oligopeptides by cell adhesion receptors. The RGD sequence, present in vitronectin, fibronectin, fibrinogen, thrombospondin, osteopontin or in the von Willebrand factor, is recognized by the integrin receptors with an α_v_ chain, e.g. α_v_β_3_ integrin [38]. These receptors are known to be associated with migration and proliferation of cells, e.g. during developmental and regeneration processes, and also during tumorigenesis [39] or vascular diseases [40]. It can be speculated that, in our system, these receptors were expressed preferentially on cells in early culture intervals (days 1–3 after seeding), when the cells were actively migrating and proliferating, while the receptors for collagen, i.e. integrins with a β_1_ chain (α_1_β_1_, α_2_β_1_ and α_3_β_1_), associated rather with a more mature state of the cells [41, 42], occurred at later culture intervals (days 3–7). In accordance with this assumption, from day 3 to day 7 there was an improvement in cell performance on surfaces with lower concentrations of Col or RGD + Col. Although the intensity of the fluorescence of talin and vinculin still remained low in the cells on these surfaces, the expression of mRNA for these proteins was similar to (vinculin) or even higher than (talin) in the cells on the control polystyrene surfaces. The cytoskeletal adaptor protein talin has been proposed to play an important role in regulating integrin affinity. Binding the talin head region to the integrin β cytoplasmic tail causes dissociation of the α and β tails, and induces a conformational change in the extracellular region that increases the affinity of an integrin receptor for its ligand [43].

Another interesting finding was that on surfaces with a lower peptide concentration the cell numbers, the spreading and the intensity of the fluorescence of talin tended to be lower on surfaces with RGD + Col than on surfaces with Col only. On samples with a higher peptide concentration, the intensity of the fluorescence of talin in the cells on RGD + Col surfaces was significantly lower than on Col surfaces. In other words, the cell adhesion was attenuated when both RGD + Col ligands were present on the material surface. Similar cell behavior was observed on combined fibronectin/collagen matrices, where the spreading and the proliferation of rat aortic smooth muscle cells was lower than on the pure fibronectin or on the pure collagen [44]. On the combined matrices, both fibronectin and collagen were recognized by the same receptor, which was probably able to receive and process the signals from both proteins simultaneously. However, a weaker cell response to this combined signal suggested steric problems of simultaneous integrin binding to both collagen and fibronectin binding sites [44]. Similar steric problems may also have occurred on our surfaces functionalized by a combination of RGD + Col.

Another possibility is that RGD and Col ligands did not both bind to the same receptor simultaneously, but that they competed for this receptor. Competition between two or more ligands for one type of integrin receptor is possible. Although a certain type of integrin receptor preferentially binds a certain ligand, this interaction is not strongly specific, and the receptor can also bind an alternative ligand [38]; for a review, see Bacakova et al. [2] This is because integrin–ligand binding depends mainly on the spatial conformation of both receptor and ligand, which is relatively flexible and changeable. For example, the receptors for RGD could also bind similar sequences, e.g. RGE, i.e. a sequence present in the Col peptide used in our study, which is shorter than RGD only by one carbon. However, similarly as in the case of combined binding of two ligands, the binding of an alternative ligand could be weaker and less bioactive, i.e. not able to initiate an adequate biochemical cascade and cell response. For example, a recombinant lipoprotein T in which RGD was mutated to RGE retained only 45 % of the binding activity for LSM 192 primary lamb joint synovial cells in comparison with lipoprotein T with intact RGD [45].

Nevertheless, the weaker response of the cells to combined RGD + Col signaling was markedly improved for samples with a higher peptide concentration. It was probably compensated by high ligand concentration and very short ligand spacing (10 nm), which was similar to the size of the head of the integrin receptor [33]. It can therefore be considered as the minimum ligand spacing. From day 3 to day 7, the cells on RGD + Col proliferated more quickly than the cells on pure RGD or on pure Col, and on day 7 they reached confluence, while on pure RGD, the cells were only semiconfluent. The expression of mRNA for talin and vinculin was highest on the RGD + Col surfaces, which was in good correlation with the highest stability of the cell adhesion on these surfaces under a dynamic load.

The highest expression of fibronectin was obtained in cells on the surface modified with RGD at the higher concentration. Similarly, on PEG hydrogels functionalized with RGD in combination with PHSRN, the production of ECM in osteoblasts was lower than in cells on surfaces bearing RGD only [46]. The explanation was that the combination of RGD + PHSRN, which mimicked ECM more closely than RGD alone, downregulated the ECM production. Similarly, the relatively long collagen peptide used in our study and its combination with RGD was able to mimic ECM more closely than RGD alone. The collagen peptide is known to bind fibronectin, which may further enhance its similarity with the native ECM. However, in our study, SPR revealed that the adsorption of fibronectin on the surfaces bearing Col and RGD + Col in both concentrations was similar as on all peptide-modified surfaces. The expected specific interactions between FN and surfaces bearing the Col peptide sequence could not be uniquely proved on the basis of the short-term SPR experiment. We hypothesize that this may be caused by the steric hindrance of the surface-bound Col peptide not allowing effective interaction with the fibronectin molecule. This is an issue that needs further investigation.

## Conclusion

It can be concluded that non-fouling PDA–PEO surfaces completely inhibited the adhesion and growth of vascular endothelial CPAE cells. Cell adhesion and growth could be restored by functionalizing these surfaces with RGD, a collagen-derived peptide QRQVVGLOGQRGERGFOGLOG-NH_2_ (Col) responsible for collagen–fibronectin interaction and a combination of these ligands in a concentration of 700 fmol/cm^2^. On surfaces with a combination of RGD + Col ligands, the stability of cell adhesion under a dynamic load was remarkably higher than on the reference polystyrene dishes, on glass coverslips and on surfaces with pure RGD or pure Col, and this stability correlated with the highest expression of talin. Thus a combination of RGD + Col at the higher peptide concentration seems to be the most appropriate way to modify biomaterials, e.g. vascular prostheses.

However, at a lower concentration of these ligands (90 fmol/cm^2^), the cell colonization in early culture intervals (day 1–3) was noticeably improved only on the RGD-bearing surfaces. On surfaces with Col or RGD + Col, this happened only at later culture intervals (day 3–7). This may be due to the expression of different types of integrin receptors in the cells in early and late culture intervals. In addition, the cell performance on surfaces with a lower peptide concentration was generally worse on the surfaces with RGD + Col than on the surfaces with Col alone. This could be explained by simultaneous or competitive binding of RGD and Col ligands to the same integrin receptor, which could alter the signal delivered to the cells and weaken the cell response.

## Electronic supplementary material

Supplementary material 1 (DOCX 23 kb)
