# Supplementary material for: Cell adhesion and growth enabled by biomimetic oligopeptide modification of a polydopamine-poly(ethylene oxide) protein repulsive surface
Source: J Mater Sci Mater Med. 2015 Oct 8;26(11):253. doi: 10.1007/s10856-015-5583-3 (PMC4598348; doi:10.1007/s10856-015-5583-3)
Supplement: Supplementary file 1 — Supplementary material 1 (DOCX 23 kb) [file 10856_2015_5583_MOESM1_ESM.docx]

**Supporting information #1**

**Preparation and characterization of the substrate materials**

α–Aminoethyl ω–alkynyl poly(ethylene oxide) (PEO) and TentaGel R Rink Amide resin were from Rapp Polymere GmbH, Tübingen, Germany; 1-ethyl 2-cyano-2-(hydroxyimino)acetate (Oxyma Pure) and Fmoc protected amino acids were from IRIS Biotech GmbH, Marktredwitz, Germany. Trifluoroacetic acid (TFA), diisopropylcarbodiimide (DIIC), piperidine and 0.01 M phosphate buffered saline pH=7.4 (PBS), fetal bovine serum (100%), tris(hydroxymethyl)aminomethane (TRIS) and fibronectin were purchased from Sigma–Aldrich, Czech Republic, and were used without further purification. Dimethylformamide (DMF) was obtained from Neratovice (Czech Republic) and was purified by distillation with ninhydrine. Sodium ^125^Iodide solution for radiolabeling was purchased from LACOMED, Czech Republic.

High–performance liquid chromatography (HPLC) and size exclusion chromatography (SEC) were performed on the gradient Knauer system with Diode Array Detection (DAD) and Alltech 3300 Evaporative Light Scattering Detection (ELSD). Analytical measurements were carried out on a Phenomenex C18, 5–μm column using gradient elution with the following solvents: A, H_2_O with 0.1% TFA; and B, CH_3_CN with 0.1% TFA. Matrix Assisted Laser Desorption/Ionization – Time Of Flight (MALDI–TOF) measurements were performed on a Bruker Biflex III mass spectrometer in reflector mode using 2.5–dihydroxy benzoic acid as the matrix in a 5:1 matrix:sample ratio. Spectroscopic ellipsometry (SE) measurements were performed on a Variable Angle Spectroscopic Imaging Auto–Nulling Ellipsometer EP³–SE (Nanofilm Technologies GmbH, Germany) with a wavelength range of λ=398.9–811.0 nm (Xe–arc lamp source, wavelength step ~10 nm) at a 70° angle of incidence. Surface Plasmon Resonance (SPR) measurements of protein adsorptions were performed with a custom–built SPR instrument (Institute of Photonics and Electronics, Academy of Sciences of the Czech Republic) utilizing the Kretschmann geometry of the attenuated total reflection method and wavelength modulation [26]. A radioassay was performed using a Bqmetr 4 ionization chamber (Empos Ltd., Czech Republic) and an NaI/Tl SpectroAnalyzer, (AccuSync Medical Research Corporation, Milford CT 06460, USA).

**Preparation of the protein–repulsive surface**

Silicon wafers were cut to 1.2 cm × 1.2 cm, and were washed by sonication in methanol and H_2_O for 15 min, along with glass slides and SPR chips. Afterwards, the wafers were subjected to chemical cleaning in 1:1:5 H_2_O_2_ (30%): NH_4_OH (25%): H_2_O at 70°C for 10 min and were dried at 100°C. Immediately prior to PDA formation, the samples were exposed to air plasma (25 W) for 5 min. A PDA anchor layer was formed on the substrate surfaces by immersing vertically oriented substrates in a dopamine solution (2 mg/mL) buffered in Tris–HCl (pH 8.5), following the procedure proposed by Lee [13]. The coated surfaces were rinsed with copious amounts of H_2_O, were sonicated in H_2_O for 15 min to remove non–specifically bound microparticles and were dried in N_2_ gas. The PDA surfaces were stabilized by thermal annealing for 24 h at 110°C before the PEO grafting procedure [22]. Pentynoyl–PEO–NH_2_ was grafted onto the PDA–modified substrates (SiO_2_/Si, glass and SPR chips) by a melt at 110°C; at this temperature, layers several microns in thickness formed from the application of ~90 μL/cm^2^ pentynoyl–PEO–NH_2_ solution (at a concentration of 8 mg/mL in methanol)[15, 16, 23, 25]. After 24 h of grafting, the substrates were rinsed with copious amounts of H_2_O, were immersed in H_2_O at 40°C for 1 h to remove any physically bound PEO, and were dried at 40°C under vacuum. The average thickness of PDA-PEO layers determined by ellipsometry was 16.2±3.1 nm for PDA and 12.7±3.9 nm for PEO respectively. The same procedure was applied for the circular 12 mm glass slides for the cell culture studies.

**Supporting information #2**

**Immunofluorescence staining**

Cells grown on the tested surfaces for 3 days were rinsed in PBS and fixed with 70% ethanol (-20^o^C, 20 min). Then the cells were pre-treated with 1% bovine serum albumin in PBS containing 0.05% Triton X-100 (Sigma, St. Louis, MO) for 20 min at room temperature, and were then incubated in primary monoclonal antibodies diluted in PBS (dilution 1:200), namely Anti-Talin, Clone 8d4 (Sigma, St. Louis, MO; Cat. No. T3287) or Anti-Vinculin, Clone hVIN-1 (Sigma, St. Louis, MO; Cat. No. V 9131). These antibodies were applied overnight at 4^o^C. After rinsing with PBS, a secondary antibody goat anti-mouse F(ab’)2 fragment of IgG conjugated with Alexa Fluor 488 (Molecular Probes, Eugene, OR; Cat. No. A11017; dilution 1:1000) was added for 1 hour at room temperature. Then the cells were rinsed twice in PBS, were mounted under microscopic glass coverslips in a Gel/Mount permanent fluorescence-preserving aqueous mounting medium (Biomeda Corporation, Foster City, CA), and were photographed under a fluorescence microscope (Olympus IX 71, Japan) equipped with a digital camera (Olympus DP 71, Japan).
